# Differential Diagnosis: Retroperitoneal Fibrosis and Oncological Diseases

**DOI:** 10.1515/med-2020-0005

**Published:** 2019-12-26

**Authors:** Antonello Sica, Beniamino Casale, Alessandro Spada, Maria Teresa Di Dato, Caterina Sagnelli, Armando Calogero, Pietro Buonavolontà, Anna Salzano, Erika Martinelli, Elisabetta Saracco, Teresa Troiani, Concetta Anna Dodaro, Dario Tammaro, Maria Luisa De Rimini, Fortunato Ciardiello, Alfonso Papa

**Affiliations:** 1 Department of Precision Medicine, University of Campania Luigi Vanvitelli, Naples, Italy; 2 Department of Pneumology and Tisiology, AO Dei Colli - V. Monaldi, Naples, Italy; 3 Pain Department, AO Dei Colli - V. Monaldi, Naples, Italy; 4 Department of Mental Health and Public Medicine, University of Campania Luigi Vanvitelli, Naples, Italy; 5 Diagnostic Service Department, AO Dei Colli - V. Monaldi, Naples, Italy; 6 Department of Advanced Biomedical Sciences, University of Naples Federico II, Naples, Italy

**Keywords:** Retroperitoneal fibrosis, Oncological disease, Lymphomas

## Abstract

Retroperitoneal fibrosis is a connective disease of the auto-inflammatory/auto-immune type of the retroperitoneum with unknown etiology and pathological mechanism. The manifestations of the pathology can be local or systemic. Amongst the local symptoms, the dull and constant pain in the hips, back or abdomen is the most frequent.

We report here a case of a 47-year-old woman, whose pathogenic mechanism could be related to an “IgG4-related disease” disorder as suggested by an increased serum level of this subclass of IgG and the positive immunohistochemistry.

The diagnosis is not easy, because this pathology generates masses; adenomegalies with retro peritoneal development, that makes it similar to lymphomas or metastases from ovarian tumors.

## 1 Introduction

Retroperitoneal fibrosis (RF) is a connective tissue disease of the autoinflammatory/ autoimmune type [1], which may be associated with systemic autoimmune diseases and systemic vasculitis [2]. The RF, named Ormond’s Disease, is a rare disease with an annual prevalence of 1/100000 that may occur in people ages between 40 and 60 years with a M / F ratio of 2: 1.

RF is characterized by growth of inflammatory and fibrous tissue in the back wall of the abdomen [3], observed in aorto-iliac bifurcation level, or atypical location (perirenal, periureteral or pelvic site) [4].

RF is idiopathic in more than 70% of cases and is secondary to several causes (infections, trauma, radiation, neoplasms, surgery, some forms of histiocytosis and drugs: ergot derivatives, bromocriptine, methyldopa, hydralazine, beta-blockers) in more than a third of cases. The idiopathic form may be limited to the retroperitoneum or involve several sites (“multifocal fibrosclerosis”) [5], which occurs with orbit pseudotumor, sclerosing cholangitis, mediastinal fibrosis and Riedel’s thyroiditis. The association with IgG4 (IgG4-related disease) has been reported [4, 5, 6, 7, 8]. The finding of elevated IgG4 clearly associates previously defined idiopathic retroperitoneal fibrosis with IgG4-Related Disease (IgG4-RD). This setting of immune diseases is characterized by the infiltration into the tissues of plasma cells expressing the immunoglobulin subclass IgG4 [6, 7, 8].

In RF cases the clinical onset may be asymptomatic, or may present general disorder to oncological or lymphoproliferative diseases [9].

The most frequent clinical presentation is a dull and constant pain in the hips, back or abdomen. Fever, fatigue, asthenia, general malaise, nausea, vomiting, anorexia, weight loss headache, arthralgias and myalgias can also occur. Inflammation rates in blood tests are commonly increased, especially in idiopathic forms.

The gold standard in therapy is to find the reduction or remission of the inflammatory / fibrotic mass or the reduction in the frequency of relapses. In spite of the lack of therapeutic guidelines, it is sure that the best prognosis can be obtained with surgical excision and/or treatment with immunomodulatory and immune-suppressive drugs like steroids, azathioprine, mycophenolate, cyclosporine, methotrexate, insulin-like growth factor-1 (IGF-1), transforming growth factor-β (TGF-β) and Tamoxifen that block progression and prevent recurrence of the disease. In some cases, the use of surgery is necessary to solve intestinal obstruction or compressive phenomena affecting other organs [3]. Few clinical cases are reported in literature on fibrosis idiopathic retroperitoneal.

The aim of this peculiar clinical case is to highlight the importance of the differential diagnosis between lymphomas and other neoplasms, and is underline the need to control the most characteristic symptom of this disease: pain, using alternative therapies.

## 2 Case report

A 47-year-old woman presented in 2017 with recurrent pelvic pain together with leg irradiation, limitation of walking and common daily activities, recurrent fever, profuse asthenia, diffuse arthromyalgia, cephalalgia syndrome and sicca syndrome. She reported an history of chronic lymphocytic thyroiditis, allergic bronchial asthma, antral gastritis, bilateral carpal tunnel syndrome, and peculiar chronic pelvic pain related to stage IV endometriosis on 07 July 2015 treated laparoscopically with abdominal-pelvic adesiolysis, removal of right ovarian cyst, salpingoplasty myomectomy, and good compensation of the algic symptomatology.

At the physician evaluation, the patient showed mild increase of inflammatory indices (ESR, PCR, alpha-2 globulins, fibrinogen), hypergammaglobulinemia, mild increase of B2 microglobulin, C4 hypocomplementemia, a positive effect of anti-thyroglobulin and anti-thyroperoxidase antibodies. ANA 1: 160, ENA positive profile for Ab anti-Ro / SSA and anti La / SSB, anti-gastric parietal cells (1:80), increased IgG4 levels (> 135 mg/dL). Appropriate screening for HIV-1,2, HCV and HBV was performed to avoid any flare-ups of viral hepatopathies during possible chemotherapy [10, 11, 12, 13, 14, 15, 16, 17, 18, 19, 20, 21, 22, 23]. She didn’t need any hepatopathies prophylaxis.

A colonoscopy examination was performed on February 27 that showed ab extrinsic compression of some colon segments with edema and mucosal congestion.

**Figure. 1:**
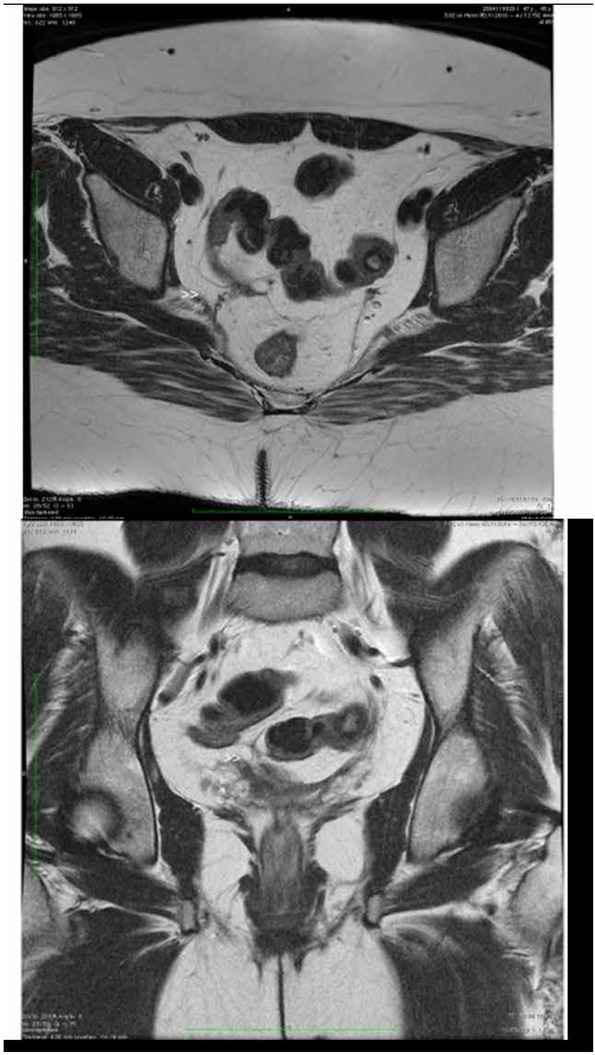
Abdomen- Pelvic MRI . Fibrotic abnormalities, with prevalence lamellar, that branch off on the right from the vaginal hemicupole, from which they reach the right ureter in the pre-bladder tract adhering to the posterior wall of the sigma 1 A - Axial T2 weighted FSE 4mm 1 B - Coronal T2 weighted FSE 4mm

In the suspected lymphoma, the patient underwent TC-PET on March 2017, which showed mild increase in fluorodeoxyglucose (FDG) uptake at the antero-superior mediastinum and at the mediastinal pre-vascular site in correspondence of lymph nodes. On 11 May 2017 she had MRI which confirmed the presence of fibrotic alterations in the context of the posterior peritoneal sheets (FIGURE 1A,1B). Therefore, an idiopathic retroperitoneal fibrosis was suspected in association with IgG4-related disease, multiple autoimmune syndrome and fibromyalgic syndrome. The patient received treatment with glucocorticoids with partial clinical remission and, subsequently, surgery on June 2017 of lower left hypogastric adhesiolysis and neurolysis of the right sacral plexus with regular postoperative course. The psychological impact before and after surgery was considerable; another laparoscopy after the operation performed two years earlier, has had a very important impact on the patient. Only the risk of a deadly pathology and the apprehension of the relatives won the fear of the surgery and its consequences.

The patient had been firstly treated with PENS Therapy on September 2017. Pens Therapy device is a monopolar electrode device designed for the electrical peripheral stimulation of peripheral nerves and peripheral nerve endings, targeted for the symptomatic treatment of peripheral chronic pain, even in areas suffering from allodynia and hyperalgesia, resistant to conventional therapies.

The procedure is painless, noninvasive, reversible and repeatable. The treatment has a duration time of 25 minutes. She had been treated with two therapies; the second one after 40 days from the first one, applying the needle always in the subcutaneous left inferior abdomen, and pain relief had been obtained with an NRS (Numeric Rating Scale) reduction from 7 to 4 after both treatments. This result had been maintained for two months until November 2017.

After the first treatment, she started oral pharmacological therapy with Tapentadol 50 mg BID, and oral endocannabinoids (described in literature as first-line treatment for analgesic therapy in neuropathic pain not responsive to opioid and/oral conventional therapy), firstly with Bedrocan 90 mg/day (Cannabis flos 19%/22% THC), then switching for side effects to Bediol 120 mg/day (Cannabis flos 6.5 % THC, 8% CBD, usually employed to avoid THC- side effects). Actually, her pain relief is 4 to 5 NRS point scale, with both, pharmacological and electrical therapy.

The patient is currently in remission and has not had any sequelae of the diagnostic intervention practiced.

At the first observation, each patient signed their informed consent for the collection of clinical data and for the use in clinical research, as established by the Ethics Committee of AOU University of Campania, Luigi Vanvitelli.

## 3 Discussion

Our case reported a rare fibrosis idiopathic retroperitoneal, peculiar for the diagnostic cues and for the inter-disciplinary management of the therapeutic-diagnostic aspects (hematologist, clinical immunologist, surgeon and anesthesiologist-resuscitator / pain therapist), compared to a relatively rare nosological entity such as fibrosis idiopathic retroperitoneal.

Our patient presented a history of allergic bronchial asthma, which suggests a preliminary immunological polarization in the Th2 sense. Hashimoto’s thyroiditis has been evaluated as indicator of immunity of multiple autoimmune disorders as shown by the positivity of ANA, ENA and anti-gastric parietal cells, which presented an autoimmune multiple syndrome (type 3 B/D) characterized by Hashimoto thyroiditis, chronic autoimmune gastritis associated with possible Sjogren’s syndrome. This is the first case that correlate RF with Sjogren type connectivitis.

The idiopathic retroperitoneal fibrosis must be considered as an autoinflammatory / autoimmune disorder of the connective tissue itself, whose pathogenic mechanism in our reported case could be related to an “IgG4-related disease” disorder as observed through increased IgG4 serum levels and the positive immunohistochemistry. The presence of a retroperitoneal mass, often associated with lymphadenopathies, makes clinicians immediately think of lymphoma or carcinoma such as ovarian cancer. These are the three most common pathologies with this radiological aspect. The differential diagnosis is extremely difficult, which is frustrating for the doctors who must face a similar case. This is further complicated if the feedback is recent without the possibility of comparison with previous examinations. The suspicion of a bulky lymphoma is founded. The pain that accompanies this pathology does not help from the diagnostic point of view.

The histological diagnosis is complex due to access to the retroperitoneal region which is only possible in laparotomy, where the diagnosis of retroperitoneal fibrosis is performed in most cases only with MRI. Often, however, biopsy or surgical exeresis are the only way to obtain a diagnosis of certainty. In particular, the surgical approach with the application of ureteral stents in case of external compression by the fibrotic mass is fundamental. This is not uncommon and is the only way to avoid renal failure. Another key point for the management of these patients is pain management. These patients have been suffering from it for years. However, it is essential to find alternative therapies to reduce the toxic effects that long-term drugs [24,25] or addiction can have.

The autoimmune component is known in retroperitoneal fibrosis, but the concomitant diagnosis of Sjogren’s syndrome is truly suggestive. Sjogren’s syndrome is usually associated with lymphoproliferative diseases.

This case demonstrates the difficulty of the diagnosis of this disease, in which in spite of the absence of surgical urgency and partial remission after glucocorticoids therapy, a biopsy was necessary to obtain a diagnosis of certainty.
